# A study of the prevalence and risk factors leading to HIV infection among a sample of street children and youth of Kathmandu

**DOI:** 10.1186/1742-6405-9-25

**Published:** 2012-08-28

**Authors:** Dibesh Karmacharya, Dongmei Yu, Sameer Dixit, Rajesh Rajbhandari, Bhawana Subedi, Sonu Shrestha, Sulochana Manandhar, Susan L Santangelo

**Affiliations:** 1 Center for Molecular Dynamics Nepal, Sawraj Sadan, Thapathali-11, Kathmandu, Nepal; 2 Massachusetts General Hospital, Psychiatric and Neurodevelopmental Genetics Unit, Center for Human Genetic Research, Boston, MA, USA; 3 Harvard Medical School, Psychiatry, Harvard School of Public Health, Epidemiology, and Massachusetts General Hospital, Psychiatric and Neurodevelopmental Genetics Unit, Center for Human Genetic Research, Boston, MA, USA

**Keywords:** HIV prevalence, Behavioural risk factors, Intravenous drug use, Street children, Kathmandu, Nepal

## Abstract

**Background:**

The true prevalence of HIV and other sexually transmitted diseases among street children in Nepal is virtually unknown while information on related behavioural risk factors in this population is non-existent. The risk of HIV infection among street children and adolescents may be especially high due to their marginalized social and economic conditions. This study was conducted to determine the prevalence of HIV infection among a sample of street children and youth of Kathmandu and to identify risk factors associated with HIV infection in this group.

A sample of street children and youth was recruited based on the purposive sampling of ten streets in Kathmandu, Nepal, known to have a high density of street children and youth. A total of 251 street children (aged 11–16 years) and youth (aged 17–24 years) were enrolled, with informed consent, from November, 2008 through June, 2009. Most of the participants (95%) were male. Case status was determined by serological assessment of HIV status; data on risk factors were obtained using structured survey interviews. HIV prevalence and rates of a number of behavioural risk factors suspected to play a role in HIV transmission among street children and youth were determined, including unprotected sex, intravenous drug use, and other risky sex and substance use behaviours.

**Results:**

Among the 251 children and youth, we found an overall HIV prevalence of 7.6%. As the sample size of females was small (n = 13) and the behavioural risk factors are likely to be quite different for boys and girls, we conducted separate analyses by gender. As our small sample of females is unlikely to be representative and lacks power for statistical testing, our report focuses on the results for the males surveyed.The strongest behavioural risk factor to emerge from this study was intravenous drug use; 30% of the male subjects were injecting drug users and 20% of those were HIV positive. Furthermore, frequency of drug injection was a highly significant predictor with a dose–response relationship; males reporting occasional injection drug use were nearly 9 times more likely to be HIV positive than never users, while weekly drug injectors had over 46 times the risk of non-users, controlling for exposure to group sex, the only other significant risk factor in the multivariate model.

**Conclusions:**

This sample of street children and youth of Kathmandu has a nearly 20-fold higher prevalence of HIV infection than the general population of Nepal (0.39%). The children and youth engage in number of high risk behaviours, including intravenous drug use, putting them at significant risk of contracting HIV and other sexually transmitted infections.

## Background

Nepal is a Low-Income Country, situated in South Asia, with a history of political and economic instability. Poverty and lack of education appear to be some of the factors that lead children and youth to leave their homes and come to the city centers in hopes of better living conditions. More often than not, however, such children are often forced to live on the street, which exposes them to many risks and dangers. Street children have been primarily grouped into three categories: children of the street, children on the street, and abandoned children [1,2]. The first category encompasses children whose entire time is spent living on the streets, including eating and sleeping. These children may have minimal contact with family or relatives. The second category describes children who spend most of the time on the streets but spend time with their families at the end of the day. Abandoned children are those who have been left to live on the streets and have no contact with their family.

The street children and youth are commonly referred to as “Khate” in Nepali, and they are known to roam around in particular areas of the city, where they engage in begging and loitering. A previous study [3] estimated the national population of street children in Nepal at about 5000; of these, 500–600 are thought to reside in Kathmandu. There has been no official census carried out on the population of street children in Kathmandu for the last decade.

There are approximately 71,250 people living with HIV and AIDS in Nepal and the estimated national HIV prevalence rate is 0.39% [4]. The risk of HIV infection among children and adolescents, especially those living on the streets, may be especially high due to their marginalized social and economic situations, as well as the existence of commercial sex and exchange sex (for food, shelter and other needs), along with intravenous drug use and other high risk behaviors in this population [5]. However, there has been no official study carried out to ascertain the actual prevalence of HIV infection in this population.

The National HIV & AIDS Strategy (2006–2011) [6] developed by the National Center for AIDS and STI Control, Government of Nepal, defines Intravenous Drug Users (IDUs), Men having sex with Men (MSM), Male Sex Workers (MSWs), Female Sex Workers (FSWs), Migrants, and Spouses of Prison inmates as the Most At-Risk Populations (MARPs) for developing HIV/AIDS. Similarly, young adults, uniformed service men and women, street children, and trafficked girls are categorized as At Risk Populations (ARPs) as per the government of Nepal, National HIV/AIDS Strategy (2006–2011) [6]. The street children and youth of Nepal may be vulnerable to sexual abuse and exploitation, such as pedophilia. The probability of these children and youth becoming involved in commercial sex and injecting drug use is high, which may increase their risk of HIV infection [5].

The true prevalence of HIV and other sexually transmitted diseases among street children in Nepal is virtually unknown, while information on behavioral risks in this population is non-existent. Worldwide, IDU accounts for 30% of HIV infection [7]. Because many of the street children and youth engage in intravenous drug use [8,9], transmission of HIV in this population is expected to be high. Unprotected sex, usually as exchange sex, is known to be common among street children worldwide [5] and may also be the case for street children and youth of Kathmandu. Furthermore, there are certain other behavioral risk factors (BRFs) which are suspected to play a role in HIV transmission among street children and youth. These include group sex, sex with male and female sex workers, anal sex, alcohol use/abuse, and glue sniffing, all of which may play a role in a street child becoming exposed to and potentially infected with HIV [10]. This study was designed to assess HIV prevalence in a sample of street children and youth from Kathmandu, as well as the BRFs associated with HIV infection in this population.

## Methods

### Study Sampling

There are an estimated 500–600 street children and youth living on the streets of Kathmandu [3]. The study sample included street children in the age range of 11–16 years, and youth in the age range of 17–24 years. A sample of 251 street children and youth were recruited for the study, based on purposive sampling frames developed within each selected cluster, targeting 10 streets of Kathmandu using information gathered through Focused Group Discussion (FGD); consultation with group leaders of the street children helped ensure an equal sampling probability across the 10 streets. Based on initial information gathered from FGDs, two to four group leaders from among the street children were selected as the main seeds. The seed leaders’ main referral list of study participants was used for random sampling. Only street children and youth in the selected areas who had lived on the streets for more than 3 months and did not return home at end of day were recruited for the study (children of the street and abandoned children). Children who were 11 years of age or less were not recruited due to concern about their ability to provide informed consent. Youth above 24 years of age who were street children and youth group leaders participated in the interview but were not recruited into the study; data of the group leaders over the age of 24 were not included in the analyses. The study team consisted of field researchers who have had experience working with the street children of Kathmandu; they were able to recruit motivators within the population of street children to encourage and facilitate the participation of study subjects.

### Data collection

The survey interviews were conducted in confidential settings by the field researchers using a questionnaire developed in the Nepali language. Data on the demographic, social, economic and living conditions of the street children and youth were collected by means of a structured KABP (Knowledge, Attitude, Behavior and Practices) survey questionnaire designed for this study. A blood sample was collected from all study subjects by trained phlebotomists and transported the same day to the laboratory facility of Intrepid Nepal Pvt., Ltd. for serological testing (November 2008-June 2009). Additional qualitative information was also gathered through FGDs conducted by trained field researchers.

The survey questionnaire and FGD guides were developed and pretested for accuracy and effectiveness in an adjoining town, which was not part of the sampling area. The tools were approved as part of the overall proposal approval process by the Nepal Health Research Council (NHRC), the ethical regulatory body of Nepal that insures the protection of human subjects in research. Written and/or verbal consent was obtained from all participants prior to the survey interview and blood sampling. Pre- and post-test counseling were done by trained counselors. The serological test results were distributed to participants in a confidential setting at a Voluntary Testing and Counseling Center (STI and AIDS Care and Treatment Services, Kathmandu, Nepal) where participants were able to receive comprehensive HIV and STI related services, if desired.

Data were collected on general demographic and family status, education, medical and health- related issues, sexual behaviors, drug injecting behaviors and other drug use, knowledge and awareness of HIV and AIDS, and media habits. Male and female participants were interviewed by investigators of the same gender. All interviews were conducted in quiet, private areas designed to make the participant feel comfortable. Altogether nine focus group discussions (FGDs) were held. Each group consisted of eight street children and youth participants. In FGDs, the participants provided information about life on the streets of Kathmandu and their knowledge and behavior related to HIV vulnerability; this greatly helped us understand the overall situation of street children and youth of Kathmandu and develop the survey questionnaire for this study.

### Serological testing

HIV serological testing was carried out using the WHO evaluated Enzyme Linked Immuno-Absorbent Assay (ELISA) (HIV1-2 ELISA Diagnostic Kit, Shanghai Kehua Bio-Engineering Co., Ltd.) at the laboratory facility of Intrepid Nepal Pvt., Ltd. Tests were performed using the WHO recommended algorithm for HIV testing and screening. All samples were tested in triplicate with ELISA. If the first test result was positive a second ELISA test was performed on that sample. If the second result was positive, further testing was not carried out, and the test result was considered “Confirmed Positive.” If the second test result yielded negative results, a third test was carried out as a “tie breaker.”

### Statistical analysis

Tests of association between the categorical/descriptive variables and HIV status were investigated via Fisher’s exact test and were applied separately in males and females; odds ratios and confidence limits were calculated to assess strength of associations. Association between the quantitative variables and HIV status was investigated by the two sample t-test and logistic regression was used to conduct multivariate analyses. In the multivariate logistic regression analyses, a fully saturated model was first established by including all the variables having a p-value < 0.1 in the univariate tests. The saturated model was then simplified by a backwards elimination stepwise procedure, using 0.05 as the p-value elimination threshold.

Because age was strongly and significantly associated with several other risk factors, we included these risk factors and excluded age itself in the fully saturated model, which resulted in a final model that included frequency of drug injection in the past month, and number of individuals involved in group sex (Table 1). All analyses were carried out using R, an open-source freely available programming language and environment for statistical computing [11]. 

**Table 1:** Multivariate analysis of high risk behaviors leading to HIV infection among the Street Children and Youth of Kathmandu, Nepal

		**OR (95% CI)**	**P**
Frequency of drug injection in last month	Never	1.0 (Ref)	
	Sometimes	8.47 (1.27, 56.35)	0.03
	Weekly	46.32 (8.19, 261.95)	<0.001
Number of individuals involved in group sex	0	1.0 (Ref)	
	2~4	6.54 (1.38, 31.01)	0.02
	> 4	0.36 (0.041, 3.23)	0.36

## Results

A total of 251 street children and youth aged 11–24 were surveyed; 19 of these subjects were positive for HIV, yielding an overall prevalence of HIV infection of 7.6% in this sample. This is over 19 times higher than the estimated HIV prevalence of the general population of Nepal at 0.39% [4].

Our sample of street children in Kathmandu consisted of mostly males (95% or 238: See Table 2). Please note that because the sample of females was so small (n = 13) and because the behavioural risk factors for girls are likely to be different from those for boys, we conducted separate analyses for girls and boys (see Table 2). The remainder of this report will focus on the results for the males surveyed, as our small sample of females is unlikely to be representative and lacks power for statistical testing.

**Table 2:** Univariate analyses on behavioural risk factors leading to HIV infection on the street children and youth of Kathmandu, Nepal

**Variable**		**Male (N=238)**				**Female (N=13)**		
		**HIV+**	**HIV-**	**Univariate**		**HIV+**	**HIV-**	**Univariate**
				**OR (CI)**	**p-value**			**p-value**
Age (mean)		21.2	15.9	1.7 (1.4, 2.0)	<0.001	22	18.5	0.13
Formal education	Yes	11	180	1.0 (Ref)		0	8	
	No	5	42	1.9 (0.50, 6.5)	0.32	3	2	0.035
Alcohol Use	No	8	55	1.0 (Ref)		0	4	
	Yes	8	167	0.33 (0.12, 0.92)	0.03	3	6	0.50
Sniffing Glue	No	6	43			0	6	
	Yes	10	179	0.40 (0.12, 1.4)	0.11	3	4	0.19
Intravenous Drug Use	No	2	164	1.0 (Ref)		1	7	
	Yes	14	57	20 (4.4, 185)	<0.001	2	3	0.51
Ever shared syringe for drug injection	No	2	167	1.0 (Ref)		1	7	
	Yes	14	53	22 (4.8, 203)	<0.001	2	3	0.51
Frequency of drug injection in last month	Never	2	164	1.0 (Ref)		1	7	
	Sometimes	3	27	9.1 (1.5, 57)	0.02	0	1	
	Weekly	10	26	32 (6.5, 152)	<0.001	2	1	0.24
Subject has regular sex partners	No	6	126	1.0 (Ref)		1	2	
	Yes	10	95	2.2 (0.70, 7.7)	0.19	2	8	1.0
Number of non-regular sex partners within a month of interview	None	6	126	1.0 (Ref)		1	2	
	1	2	15	2.8 (0.52, 15)	0.23	0	2	
	> 1	8	80	2.1 (0.70, 6.3)	0.18	2	6	1.0
Subject participates in group sex	No	9	141	1.0 (Ref)		0	7	
	Yes	7	81	1.4 (0.41, 4.3)	0.60	3	3	0.070
Frequency of anal sex	Never/Rarely	10	138	1.0 (Ref)		1	5	
	Sometimes/Frequently	6	84	0.99 (0.28, 3.1)	1	2	5	1.0

Data for this integarted bio-behavioral HIV study were collected from 251 street children and youth of Kathmandu, Nepal from November, 2008 through June, 2009.

Nearly half the male children and youth interviewed (48%) had been living on the streets for more than 5 years and the majority had living parents (63%), while some (11%) reported that both parents were deceased. Nearly half the male participants (48%) left home when they were between 6 and 11 years old, while 24% were younger than 6 when they left home. Some level of formal education had been attained by 80% of the male children and youth interviewed (and only 62% of the females), with 14% of the males reporting to have studied until at least grade 5. Among boys with no formal education, HIV prevalence (11%) was significantly higher than among those with some education (5.8%). It is noteworthy that among the 5/13 girls with no formal education, 60% (3/5) were HIV positive, while none of the 8 girls with some education were infected.

The vast majority of the male children and youth (94%) reported to have had vaginal intercourse; 64% of those boys had their first sexual intercourse between the age of 12 and 16 years while 24% first experienced sexual intercourse before age 12. Anal sex was experienced by 39% of the boys and 61% of the boys interviewed had had sex with a female sex worker while 12% had had sex with a male sex worker. Furthermore, 37% of the boys had participated in group sex, with some (32%) engaging in group sex with four or more participants. Many of the street children admitted to having sexual partners other than their regular partners (44% of male participants), and more than 37% of the boys reported having had sexual intercourse with more than 1 partner other than their regular partner in the last month alone. Regular use of condoms appeared to be low in the majority of the street children and youth who were sexually active, with 59% responding that they did not regularly use condoms during sexual intercourse. A total of 87% of the boys reported they had not used condoms during their last sexual encounter despite the fact that 97% reported to have heard of HIV/AIDS, and 91% reported to understand that unsafe sexual practices can lead to HIV infection (data not shown). The vast majority (91%) interviewed stated that they were aware such unsafe sexual practices put them at increased risk of contracting HIV. However, none of these sexual behaviours significantly discriminated between cases with HIV infection and non-cases.

Thirty percent of this sample of male street children (and 38% of the females) were self-reported intravenous drug users and 20% (14/71) of male IDUs were HIV positive (p < 0.001), (as were 2/5 or 40% of female IDUs) (Table 2). Boys reporting occasional injection drug use were 9 times more likely to be HIV positive than never users, while weekly drug injectors were at 32 times the risk of non-users. Among the male IDUs, 28% shared needles with their peers and 21% of those sharing needles were HIV positive (OR = 21.7; p < 0.001).

A significant, but unexpected association was identified between alcohol consumption and HIV status among the boys in the univariate analyses. Alcohol use appeared to be protective, conferring about 1/3 the risk of HIV infection as compared to non-drinkers. Alcohol use was uncorrelated with IDU (data not shown).

### Multivariate analysis

In the final multivariate model, which included only the data on boys, number of individuals involved in group sex was a significant risk factor, controlling for drug injection frequency, and was associated with a 6.5-fold increased risk for the boys who endorsed having 2–4 group sex partners as compared with boys who did not participate in group sex.

Intravenous drug use remained a very strong risk factor in the multivariate analyses. The odds of HIV infection were nearly 9 times greater (OR = 8.5; 95% CI: 1.3, 56.4) among boys who ‘sometimes’ injected drugs compared to those who never injected, adjusted for number of group sex partners, while the risk increased to over 46 times that of non IDUs (OR = 46.3; 95% CI: 8.2, 262) for the boys who injected drugs at least weekly, adjusting for group sex exposure. Alcohol use was not a significant predictor of risk in the multivariate model.

## Discussion

This study, the first of its kind in Nepal, was designed to a) assess the HIV status of a pilot sample of street children and youth of Kathmandu and b) identify risk factors associated with HIV infection among these children and youth. The serological tests revealed an HIV prevalence rate of nearly 8% among the sample of street children and youth surveyed. This is nearly 20 times greater than the estimated HIV prevalence of 0.39% for the general population of Nepal [4]. A similar study carried out in India reported a prevalence rate for HIV infection of 1% in street children of Kolkata [12].

We found significant associations between HIV status and intravenous drug use, and sharing needles. Furthermore, frequency of drug injection was a highly significant predictor with a dose–response relationship; while the risk of HIV infection increased nearly 9-fold among those who occasionally injected drugs, it rose to more than 46 times the baseline risk of those who never engaged in IV drug use for those injecting on at least a weekly basis. These results are similar to findings from other studies where IDUs have been statistically linked to HIV [13]. However, this sample may be unique in that, unlike many/most studies of HIV risk factors for children and youth, it was characterized by a fairly high prevalence of drug injection behaviour, involving 30% of boys and 38% of girls.

In addition, both age and number of group sex partners were found to be independent risk factors for HIV infection among this sample of street children of Kathmandu. As noted above, and in Table 3, age was both significantly associated with the outcome, HIV infection, and significantly associated with several of the other putative risk factors, or exposures, thus meeting the requirements for the classical definition of a confounder, thus we cannot rule out the possibility that age may act as a modifier of some of these other risk factors. Boys engaging in group sex with 2–4 partners carried a more than 6-fold increased risk for HIV infection.

**Table 3:** Associations between Age and Other HIV related risk behaviors among Street Children and Youth of Kathmandu, Nepal

**Risk behaviors**		**Age (mean)**	**p-value**
Formal education	Yes	15.98	
	No	17.23	0.03
Whether have regular sex partners	No	15.78	
	Yes	16.75	0.02
Number of non-regular sex partners within a month of interview	None	15.78	
	1	16.53	
	> 1	16.80	0.070
Group sex	No	15.87	
	Yes	16.84	0.03
Frequency of anal sex	Never/Rarely	16.29	
	Sometimes/Frequently	16.12	0.71
Alcohol use	No	16.14	
	Yes	16.26	0.82
Sniffing Glue	No	18.12	
	Yes	15.74	<0.001
Intravenous Drug Use	No	15.52	
	Yes	17.87	<0.001
Ever shared syringe for drug injection	No	15.51	
	Yes	18.03	<0.001
Frequency of drug injection in last month	Never	15.52	
	Sometimes	16.83	
	Weekly	18.72	<0.001

p-value, generated by a t-test, reflects significance of association between Mean Age and Risk behavior catogories.

### Potential Weaknesses of the Study

We have concerns regarding the validity of some of the answers given by the children on questions that were especially sensitive; the children may not have always provided accurate responses, as the stigma associated with HIV is high. A study carried out on children affected by AIDS in Nepal reported that children reported increased fear and discomfort associated with their HIV status being known in the community [14]. As a result, it may be assumed that street children and youth may not always have been forthright when answering sensitive questions, such as those concerning condom use and anal and group sex. This may be one reason why this study did not find a clear correlation between condom use or anal sex and HIV, although unprotected anal sex with multiple sex partners has been linked to increased incidence of HIV in adults in prior research [15].

#### Program Intervention

The concerned stakeholders, including UNICEF/Nepal, were presented with the findings of the study. Participating NGOs and government agencies that have harm reduction programs targeted to street children and youth participated in the dissemination event. Some of the NGOs pledged to work on HIV prevention and awareness in this population, including the initiation of HIV service oriented programs.

## Conclusions

This sample of street children and youth of Kathmandu has a nearly 20-fold higher prevalence of HIV infection than the general population of Nepal (0.39%). The children and youth engage in a number of high risk behaviours, including intravenous drug use and sharing needles, putting them at a significant risk of contracting HIV and other sexually transmitted infections. Lack of formal education was a risk factor, albeit not the strongest one, highlighting the need for more education and prevention outreach in a population where formal education is not compulsory and illiteracy is even now, not uncommon.

If this male sample can be used as an indicator of risk among the rest of the street children and youth of Kathmandu, then many of these children are vulnerable and at high risk for HIV infection. Among the most vulnerable street children and youth, those who inject drugs and share needles, there is a danger of increased prevalence of HIV infection without targeted interventions for this at-risk group. Although a much larger study would be required for a more comprehensive picture of the problem throughout Nepal, we hope the findings of this study will raise awareness among concerned health policy makers and ultimately lead to effective intervention programs to address the high prevalence of HIV infection among the street children and youth of Kathmandu before it becomes an epidemic.

## Abbreviations

AIDS: Acquired Immune Deficiency Syndrome; ARP: At Risk Population; BRF: Behavioural Risk Factors; ELISA: Enzyme Linked Immuno-Absorbent Assay; FGD: Focused Group Discussion; FSW: Female Sex Worker; HIV: Human Immunodeficiency Virus; IDU: Intravenous Drug Use; KABP: Knowledge Attitude, Behaviour and Practices; MARP: Most At Risk Population; MSW: Male Sex Worker; NHRC: Nepal Health Research Council; OR: Odds Ratio; STI: Sexually Transmitted Infection; WHO: World Health Organization.

## Competing interests

The authors declare no conflicts of interest related to this study.

## Authors’ contributions

The overall study was designed by DK, SD, RR and BS with input from SLS. The field data collection was led by BS and supported by RR and SD. The lab work was done by SM under the supervision of SS. DY carried out all data analyses; DY and SLS interpreted results of statistical testing and made inferences. The manuscript was prepared by DK, SLS, DY and SD. All authors read and approved the final manuscript.

## References

[B1] Barker G Knaul F Exploited entrepreneurs: Street and working children in developing countries 1991 Childhope: Working Paper 1

[B2] Campos RRM Ude W Social networks and daily activities of street youth in Belo Horizonte, Brazil. Street Youth Study Group Child Dev 1994 65 319 330 10.2307/1131386 8013224

[B3] Child Workers in Nepal Concerned Centre (CWIN) HIV/AIDS and Street Children archived report. ref. CAR-60 2579 2002 Kathmandu: CWIN

[B4] National Estimates of HIV infections, 2009 Nepal National Estimates of HIV infections, 2009 2010 Nepal. Kathmandu: National Center for AIDS and STD Control (NCASC)

[B5] Towe VL ul H Zafar ST Sherman SG Street life and drug risk behaviors associated with exchanging sex among male street children in Lahore, Pakistan J Adolescent Health 2009 44 222 228 10.1016/j.jadohealth.2008.09.003 PMC2671069 19237107

[B6] National HIV/AIDS Strategy (2006–2011) 2007 Kathmandu: National Center for AIDS and STD Control (NCASC)

[B7] Report on the global AIDS epidemic 2008 UNAIDS

[B8] Dmirty MK Lauren Z HIV seroprevalence in street youth, St Petersburg, Russia AIDS 2007 21 2333 2340 10.1097/QAD.0b013e3282f125b3 18090282

[B9] Robbins CL Zapata L Multicity HIV seroprevalence in street youth, Ukraine Int J STD AIDS 2010 21 489 496 10.1258/ijsa.2010.010097 20852199

[B10] Rooyen L Hartell CG The health of the street child - The whole says more a synergy of research Int J Adolescence and Youth 2001 10 297 317

[B11] R Foundation for Statistical Computing A language and environment for statistical computing, reference index version 2.x.x 2005 Vienna: 14: Development Core Team

[B12] Bal BMR Mallick AH Chakraborti S Sarkar K Nontobacco substance use, sexual abuse, HIV, and sexually transmitted infection among street children in Kolkata, India Substance Use Misuse 2010 45 1668 1682 10.3109/10826081003674856 20590379

[B13] Kral AH BR Booth RE Watters JK HIV seroprevalence among street-recruited injection drug and crack cocaine users in 16 US municipalities Am J Public Health 1998 88 108 113 10.2105/AJPH.88.1.108 9584014 PMC1508387

[B14] A Situation Assessment of Children Affected by AIDS in Nepal 2009 Kathmandu: NCASC/Save the Children-Nepal/CREHPA

[B15] Koblina BA HM Colfaxc G Risk factors for HIV infection among men who have sex with men AIDS 2006 20 731 739 10.1097/01.aids.0000216374.61442.55 16514304

